# Performance comparison of EasyFix G26 and HYDRASYS 2 SCAN for the detection of serum monoclonal proteins

**DOI:** 10.1002/jcla.23254

**Published:** 2020-03-06

**Authors:** Fatimah Diana Amin Nordin, Mohd Khairul Nizam Mohd Khalid, Siti Mastura Abdul Aziz, Nor Aina Mohamad Bakri, Siti Nurwani Ahmad Ridzuan, Julaina Abdul Jalil, Anasufiza Habib, Yusnita Yakob

**Affiliations:** ^1^ Molecular Diagnostics and Protein Unit Institute for Medical Research Kuala Lumpur Malaysia; ^2^ Biochemistry Unit Institute for Medical Research Kuala Lumpur Malaysia

**Keywords:** EasyFix G26, HYDRASYS 2 SCAN, immunofixation electrophoresis, serum monoclonal protein, serum protein electrophoresis

## Abstract

**Background:**

Serum protein electrophoresis (SPE) is a widely used laboratory technique to diagnose patients with multiple myeloma (MM) and other disorders related to serum protein. In patients with MM, abnormal monoclonal protein can be detected by SPE and further characterized using immunofixation electrophoresis (IFE). There are several semi‐automated agarose gel‐based systems available commercially for SPE and IFE. In this study, we sought to evaluate the analytical performance of fully automated EasyFix G26 (EFG26) and semi‐automated HYDRASYS 2 SCAN (H2SCAN) for both SPE and IFE.

**Methods:**

Both instruments were operated according to manufacturer's instructions. Samples used include a commercially available normal control serum (NCS) and patients' specimens. The following were evaluated: precision and comparison studies for SPE, and reproducibility and comparison studies for IFE. Statistical analyses were performed using Microsoft Excel.

**Results:**

For SPE repeatability study, our results showed that EFG26 has higher coefficient of variation (%CV) compared with H2SCAN for both samples except for monoclonal component with %CV of 0.97% and 1.18%, respectively. Similar results were obtained for SPE reproducibility study except for alpha‐1 (4.16%) and beta (3.13%) fractions for NCS, and beta fractions (5.36%) for monoclonal sample. Subsequently, reproducibility for IFE was 100% for both instruments. Values for correlation coefficients between both instruments ranged from 0.91 to 0.98 for the five classic bands.

**Conclusion:**

Both instruments demonstrated good analytical performance characterized by high precision, reproducibility and correlation.

## 1 INTRODUCTION

Serum protein electrophoresis (SPE) is an established technique used for screening abnormal protein known as monoclonal proteins (M proteins, paraproteins, monoclonal immunoglobulins) in the serum.^1^ SPE aids in the diagnosis of patients with multiple myeloma (MM) and other serum protein disorders. In more than 80% of patients, abnormal proteins can be detected by using SPE technique.^2^ Based on the principle of zone electrophoresis, SPE separates the serum protein into five major fractions (albumin, alpha‐1 globulins, alpha‐2 globulins, beta globulins, and gamma globulins) which primarily according to their charge at a given pH. As a result, these fractions defined the pattern of SPE. In comparison with normal serum pattern, patient with MM usually produced an abnormal serum pattern which can be evaluated visually on SPE gel and also by electropherograms that were generated by the densitometer.

The confirmation of the presence and identification of monoclonal protein type can be accomplished by immunofixation electrophoresis (IFE); a “gold standard” method which consists of an electrophoresis phase and a fixation phase. Fixation phase allows a protein to be anchored after electrophoresis phase by forming an insoluble complex between antiserum containing anti‐IgG, anti‐IgA, anti‐IgM, anti‐light chain kappa, and anti‐light chain lambda with specific antibody in the serum therefore allowing the protein to be characterized.^3^, ^4^, ^5^

Since year 2008 our laboratory has been using a semi‐automated HYDRASYS 2 SCAN (H2SCAN) to perform SPE and IFE test for diagnostic purposes. The availability of a fully automated instrument raised an interest for us to compare the performance between both systems. There was a study between both instruments published by Napodano et al in 2017, but only on the comparison of protein immunofixation electrophoresis.^6^ To our knowledge, this is the first performance comparison study that has been performed by using EasyFix G26 (EFG26) which claimed to be the first instrument that could perform IFE fully automatically. Therefore, the aim of this study was to evaluate the analytical performance of a fully automated system, EFG26 with H2SCAN.

## 2 MATERIALS AND METHODS

### 2.1 Gel electrophoresis using easy fix G26

Fully automated agarose gel SPE and IFE were performed according to the manufacturer's instructions using the EFG26 (Interlab) with SRE636K and SRE628K kits (Interlab), respectively. EFG26 automatically performed all the procedures for SPE which include the application of samples on agarose gel plate; electrophoretic migration (pH 8.9 at 220 V and 20°C); gel denaturation; gel staining (with Acid Blue); gel destaining; gel drying; and densitometric reading using Elfolab™ densitometer (Interlab).

Procedures for IFE were also fully automated beginning from dispensation/dilution of samples, followed by application of samples on agarose gel plate; electrophoretic migration (380 V at 20°C); application of the fixative solution and antisera (IgG, IgA, IgM, kappa:κ, or lambda:λ); gel incubation; gel blotting; gel denaturation; gel washing; gel staining (with acid violet); gel destaining; and final gel drying (80°C).^7^

### 2.2 Gel electrophoresis using HYDRASYS 2 SCAN

Semi‐automated SPE and IFE were performed using the H2SCAN (Sebia) according to the manufacturer's instructions with Hydragel protein (E) 15/30 gel and Hydragel IF 2/4 gel (Sebia), respectively. SPE was manually started with the application of 10 µL sample to the sample template followed by the placement of the template into the migration compartment. The procedures continued automatically with electrophoretic migration (pH 9.1 at 20 W and 20°C) and gel drying (65°C). Subsequently, the gel was transferred manually to the staining compartment and followed by automated procedures which involve gel staining (with amido black), gel destaining, and gel drying. Lastly, the gel was transferred to the scanning compartment and scanned with Phoresis densitometer (Sebia).

Meanwhile for IFE, the automated procedures include sample application; electrophoretic migration (20 W at 20°C); incubation with fixative solution and antisera (IgG, IgA, IgM, κ, or λ); gel drying; gel staining (with acid violet); gel destaining; and gel final drying (50°C), whereas the manual steps include handling of the samples and gel, application of fixative as well as antisera, and setting up the instrument for operation.^8^

For the detection of monoclonal bands, visual inspection of stained gels was also performed for both instruments apart from densitometric reading.

### 2.3 Analysis and evaluation

Quantitative results of SPE were evaluated through precision studies which involve repeatability (*within‐run precision*) and reproducibility (*day‐to‐day imprecision*). Repeatability for SPE (n = 30) was analyzed based on CLSI guideline EP15‐A2 and Ministry of Health Malaysia guideline using a commercially available normal control serum (NCS; manufactured by Sebia) and patient's monoclonal sample.^9^, ^10^ Reproducibility for SPE (n = 15) was also evaluated using NCS and patient's monoclonal sample for five different days based on CLSI guideline EP10‐A2 and Ministry of Health Malaysia guideline. Linear regression analysis for SPE was performed between the two instruments through comparison study using patients’ samples (n = 50) submitted for routine SPE test and the statistical significance was set at *P* < .05. The agreement between both instruments was later assessed by Bland‐Altman analysis. Meanwhile for IFE, reproducibility study was done over a period of 3 days (n = 4) and the agreement between the two instruments was also observed (n = 4). Statistical analyses were performed using Microsoft Excel.

## 3 RESULTS

### 3.1 Precision studies for serum protein electrophoresis

In the repeatability study, 30 aliquots of NCS and monoclonal sample were assayed by both EFG26 and H2SCAN. As revealed by NCS, our findings for the five classic bands expressed as coefficients of variation (%CV) ranged from 3.35% to 11.86% for EFG26 compared with 0.82%‐5.37% for H2SCAN (Table 1: a). Meanwhile for monoclonal sample, the %CV ranged from 0.97% to 14.38% for EFG26 and 1.18%‐10.54% for H2SCAN (Table 1: b).

**Table 1:** Repeatability (*within‐run precision*) and reproducibility (*day‐to‐day imprecision*) Studies on NCS and monoclonal sample for SPE

Instrument	EFG26			H2SCAN		
Fraction	Mean (%)	SD (%)	CV (%)	Mean (%)	SD (%)	CV (%)
(a) Repeatability (*Within‐run precision*): Normal Control Serum (NCS; n = 30)						
Albumin	53.17	1.78	3.35	64.57	0.53	0.82
Alpha 1	3.30	0.39	11.86	2.91	0.16	5.37
Alpha 2	12.74	0.58	4.55	9.97	0.17	1.68
Beta	15.10	0.62	4.09	10.59	0.27	2.57
Gamma	15.69	0.95	6.02	11.95	0.47	3.97
(b) Repeatability (*Within‐run precision*): Monoclonal Sample (n = 30)						
Albumin	41.52	3.55	8.56	49.13	0.72	1.46
Alpha 1	2.23	0.32	14.38	2.14	0.23	10.54
Alpha 2	11.11	0.72	6.47	9.45	0.21	2.25
Beta	10.47	0.87	8.31	6.91	0.13	1.95
Gamma	34.52	2.69	7.80	32.38	0.68	2.10
*Monoclonal*	27.36	0.26	0.97	27.22	0.32	1.18
(c) Reproducibility (*Day‐to‐day imprecision*): Normal Control Serum (NCS; n = 15)						
Albumin	55.79	1.42	2.54	65.89	0.77	1.17
Alpha 1	2.97	0.12	4.16	2.45	0.12	5.08
Alpha 2	11.95	0.42	3.48	9.50	0.20	2.11
Beta	14.14	0.44	3.13	10.27	0.33	3.24
Gamma	15.15	0.69	4.58	11.86	0.45	3.83
(d) Reproducibility (*Day‐to‐day imprecision*): Monoclonal Sample (n = 15)						
Albumin	26.57	1.36	5.11	25.37	0.54	2.13
Alpha 1	1.52	0.25	16.68	1.73	0.16	9.12
Alpha 2	5.42	0.35	6.44	5.91	0.21	3.53
Beta	4.86	0.26	5.36	4.36	0.28	6.51
Gamma	61.63	1.66	2.69	62.63	0.69	1.10
*Monoclonal*	59.42	0.40	0.67	59.62	0.27	0.45

For reproducibility study, 15 aliquots of both NCS and monoclonal sample were also assayed by both instruments. The %CV for NCS ranged from 2.54% to 4.58% for EFG26 compared with 1.17%‐5.08% for H2SCAN (Table 1: c). Also, for the monoclonal sample, the %CV ranged from 0.67% to 16.68% for EFG26 compared with 0.45%‐9.12% for H2SCAN (Table 1: d).

### 3.2 Comparison study for serum protein electrophoresis

**Figure 1:**
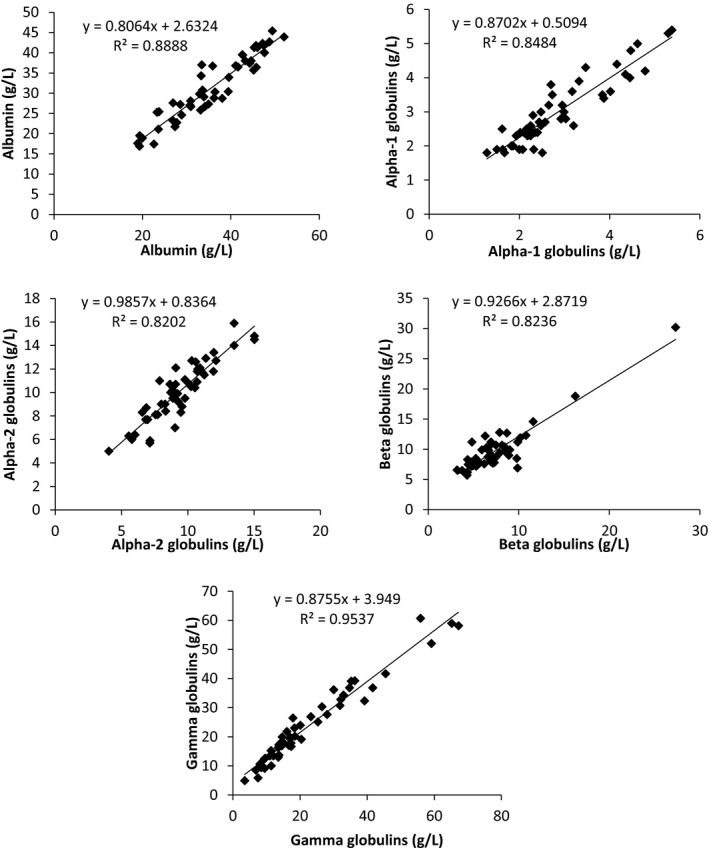
Linear correlation between H2SCAN (*x*‐axis) and EFG26 (*y*‐axis) for the five fractions with correlation coefficients (*r*) of .94, .92, .91, .91 and .98 for the albumin, alpha‐1 globulins, alpha‐2 globulins, beta globulins, and gamma globulins with *P* value <.001, respectively

Fifty different serum samples were analyzed by both instruments for SPE. The five major serum fractions were quantified and linear regression plots were produced as shown in Figure 1. Correlation coefficients (*r*) for the five classic bands showed strong correlation between the two instruments with values close to 1. The values were 0.94, 0.92, 0.91, 0.91, and 0.98 for the albumin, alpha‐1 globulins, alpha‐2 globulins, beta globulins, and gamma globulins, respectively (Figure 1).

**Figure 2:**
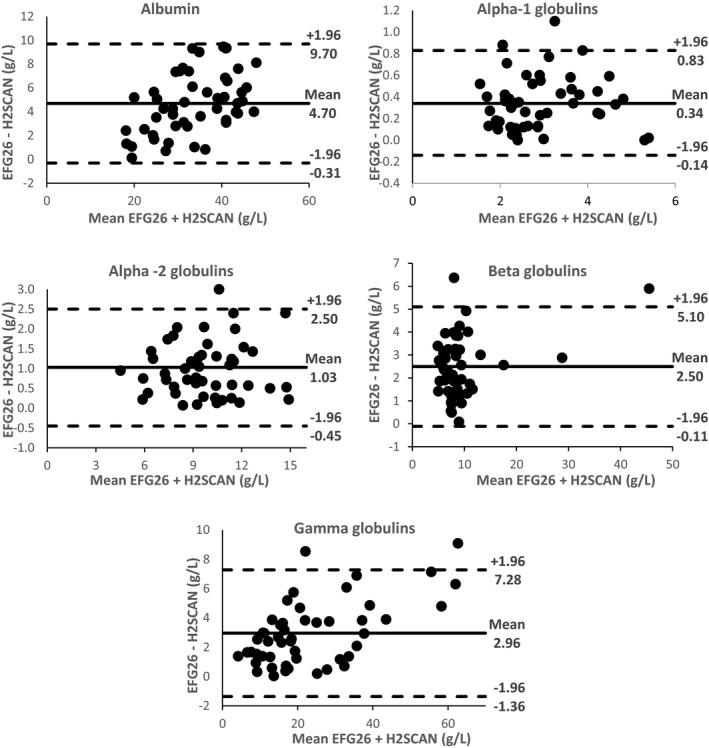
Bland‐Altman analysis of each fraction by H2SCAN vs EFG26. The mean ± 2 SD (g/L) between the instruments is reported in each graph

Results of Bland‐Altman analyses demonstrated excellent concordance between EFG26 and H2SCAN (Figure 2 and Table S1). The central 0.95 interval (mean difference ± 2 SD) indicated a good agreement between both instruments for all fractions which 95% of the data points were found within ± 2S of the mean difference with *P* value <.001.

### 3.3 Reproducibility study for immunofixation electrophoresis

**Figure 3:**
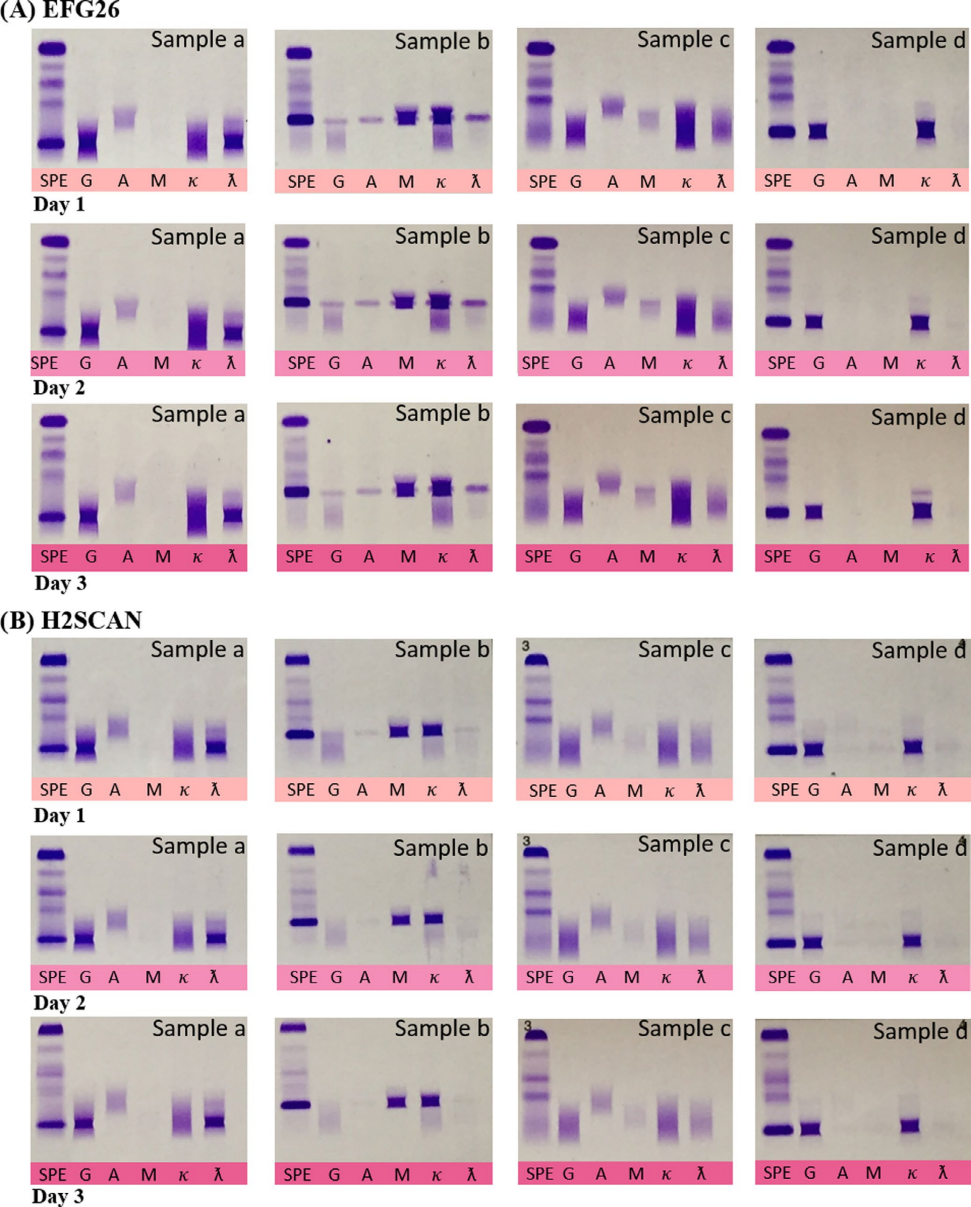
In the reproducibility study, the immunofixation revealed an IgGλ monoclonal protein (Sample a), IgMκ monoclonal protein (Sample b), no monoclonal component (Sample c), and IgGκ (Sample d) by both instruments. A) Immunofixation gels for Day 1, Day 2, and Day 3 obtained from EFG26. B) Immunofixation gels for Day 1, Day 2, and Day 3 obtained from H2SCAN

Four serum samples were analyzed on both systems to describe the closeness of agreement of results under changing conditions for a period of three days. The results of the reproducibility study were 100% for both instruments which can be visualized in Figure 3. The IFE revealed an IgGλ monoclonal protein (Sample a), IgMκ monoclonal protein (Sample b), no monoclonal component (Sample c), and IgGκ monoclonal protein (Sample d) for both instruments. As for visual inspection, the resolution of the gel can be seen higher with EFG26 but the clarity and sharpness of the bands were better with H2SCAN. Generally, there were no differences between EFG26 and H2SCAN with respect to the characterization of monoclonal proteins.

### 3.4 Comparison study for immunofixation electrophoresis

**Figure 4:**
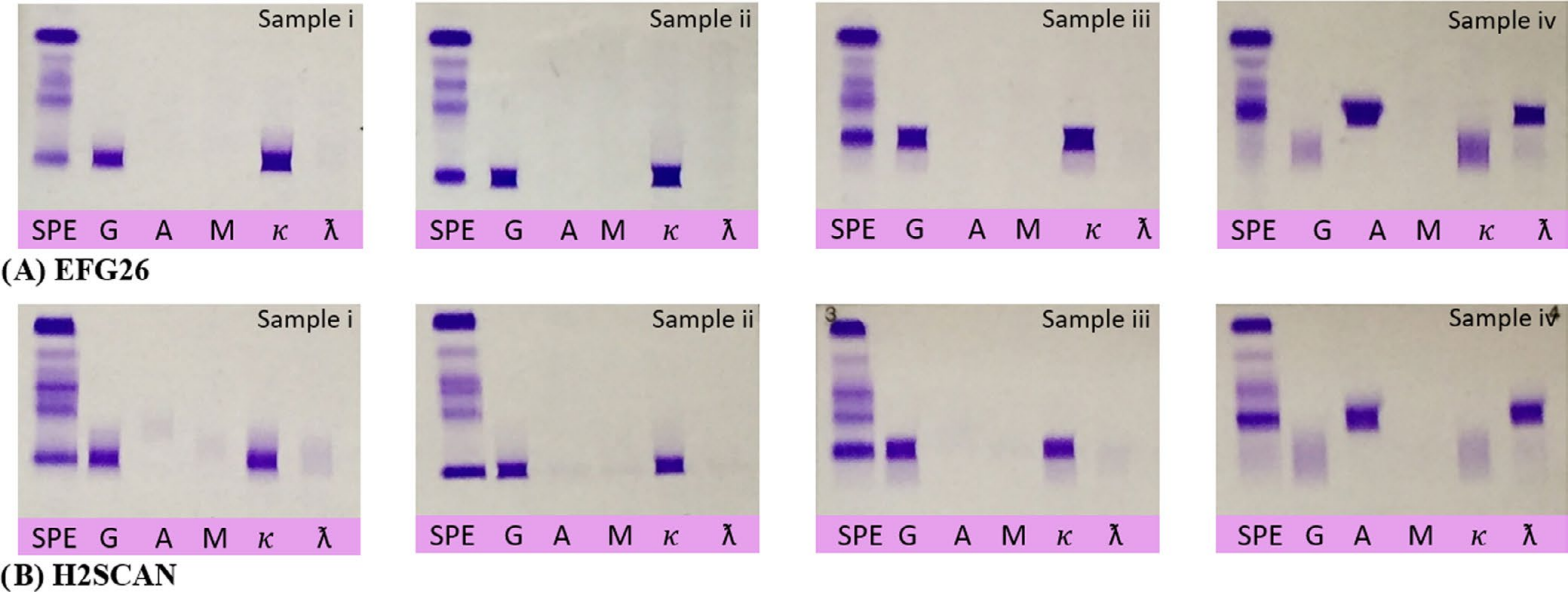
In the comparison study, immunofixation revealed an IgGκ monoclonal protein for Samples i, ii, and iii and IgAλ for Sample iv by both instruments. A) Immunofixation gel obtained from EFG26. B) Immunofixation gel obtained from H2SCAN

Four serum samples were analyzed on both systems to measure the agreement between EFG26 and H2SCAN. A 100% agreement was observed between the two instruments which can be visualized in Figure 4. The IFE revealed an IgGκ monoclonal protein (Samples i, ii, and iii) and IgAλ monoclonal protein (Sample iv) for both instruments. Altogether, there were also no differences between EFG26 and H2SCAN with respect to the characterization of monoclonal proteins. The resolution was also higher with EFG26, and the clarity of the bands was better with H2SCAN.

## 4 DISCUSSION

**Figure 5:**
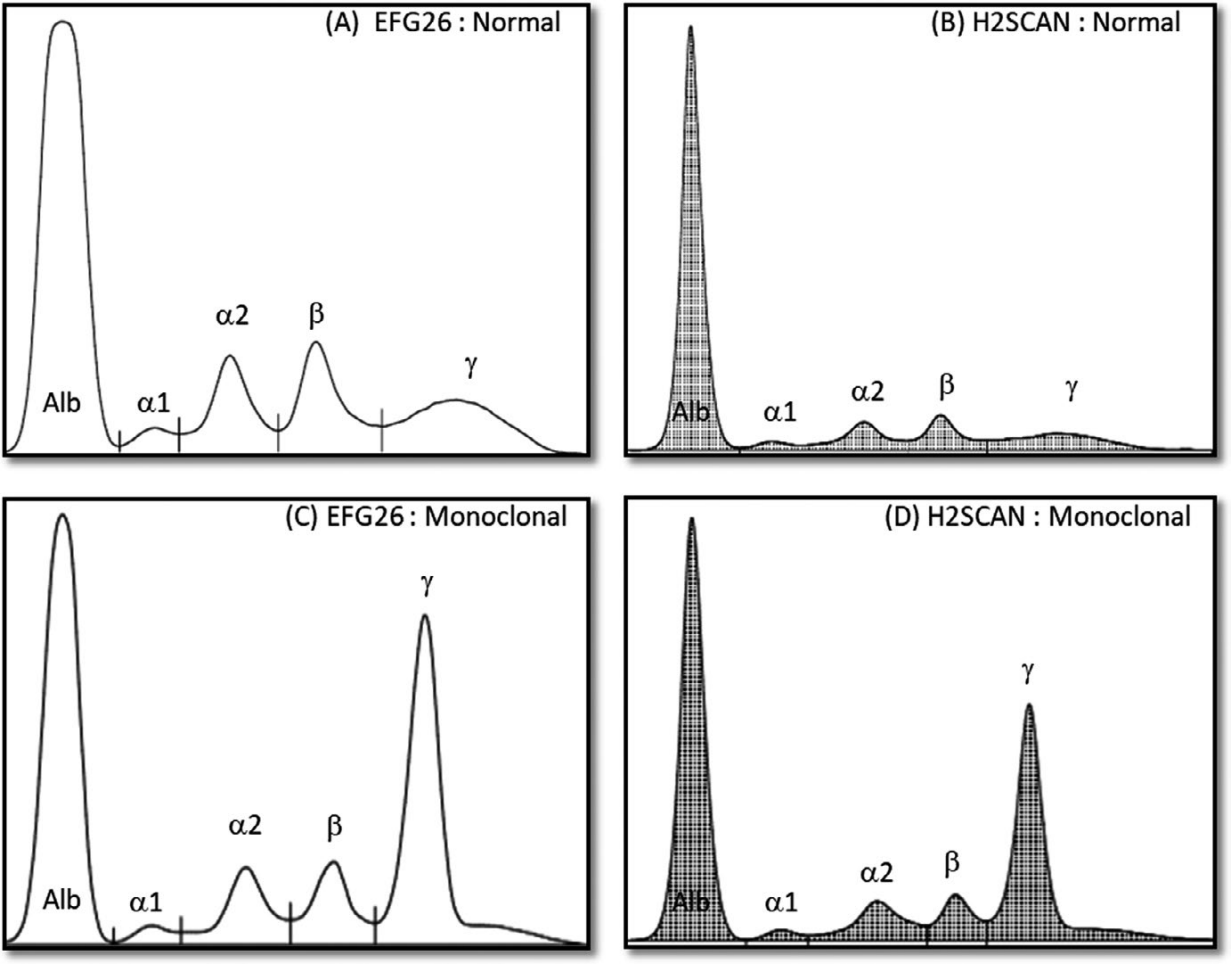
Electropherograms of a healthy serum sample obtained by (A) EFG26 and (B) H2SCAN with five major serum protein fractions. These followed by representations of an abnormal serum sample by (C) EFG26 and (D) H2SCAN with monoclonal protein, creating a large spike in the gamma region. (Alb: albumin; α1: alpha‐1 globulins; α2: alpha‐2 globulins; β:beta globulins; and γ:gamma globulins)

An instrument with a good performance is critically important in detecting the presence of serum monoclonal protein as it aids in the diagnosis of MM. In SPE, screening procedure initially started with the identification of monoclonal bands by visual examination of the gel. This visual examination is very subjective and operator dependent. Electropherogram obtained by densitometric scanning of SPE gel aids in the monoclonal detection by providing quantitative figures for each protein fractions. The generated results are widely accepted by the clinicians in reviewing the sample.^11^ In this study, both EFG26 and H2SCAN electropherograms produced five major serum protein fractions from a healthy serum sample and from a patient with monoclonal protein (Figure 5).

Assessment of precision is part of a process of verifying or validating a method which confirms the suitability for use.^12^ Overall, EFG26 produced a higher %CV values compared with H2SCAN in both repeatability and reproducibility studies for the classic bands. As for monoclonal component, EFG26 produced a lower %CV value (0.97%) in the repeatability study and a higher %CV value in the reproducibility study (0.67%) compared with H2SCAN which were 1.18% and 0.45%, respectively.

The %CV values obtained by both instruments were comparable to the %CV values reported by Kahn and Strony who performed their study using cellulose acetate electrophoresis. The study showed that the precision of densitometric quantification fraction %CV ranged from 1.3% to 5.1% for albumin, 4.4%‐20.2% for alpha‐1 globulins, 1.8%‐9.2% for alpha‐2 globulins, 1.6%‐14.0% for beta globulins, and 2.5%‐27.2% for gamma globulins.^13^ In addition, a review by Bossuyt on protein fractionation using capillary method by Paragon 2000^®^ and Capillary^®^ also showed similarity with both %CV values obtained for the five classic protein fractions were consistently <8% for intra‐run (repeatability) and <11% for between‐run precision (reproducibility) studies. Their overall precision was 1.2% for albumin, 6.1% for alpha‐1 globulin, 3.2% for alpha‐2 globulin, 3.2% for beta globulin, and 4.3% for gamma globulin fractions.^14^ Furthermore, EFG26%CV values for NCS also have a comparable performance. Therefore, the values obtained in our study were in accordance with those of the literature with both systems provided highly repeatable and reproducible results.

As with our comparison study, the results showed high correlation and good agreement between the two instruments. The highest correlation coefficient (*r*) was for the gamma globulins (.98) (*r* between .96 and .99 in other studies), whereas the lowest correlation coefficient were obtained for both alpha‐2 and beta globulins (.91) (*r* between .73 and .92 in other studies).^14^ These findings were in concordance with another correlation study performed by Chartier et al between two automated capillary electrophoresis systems which are between the Capillarys 2^®^ (Sebia) and V8^®^ (Helena) with Hydrasys‐Hyrys^®^ (Sebia). The study revealed a closely correlated results among the three instruments with r values ranged from 0.81 to 0.96 (five fractions).^15^

In conclusion, both EFG26 and H2SCAN provide acceptable precision, can be reproducible, and produce accurate identification of monoclonal protein. EFG26 has the advantage of easy to use but with slightly higher %CV values. Nevertheless, both instruments have good analytical performances with good correlation. Therefore, both instruments are suitable to be used for the detection of monoclonal proteins in patients with MM.

## ETHICS APPROVAL

This study protocol was checked and approved by Medical Research & Ethics Committee (MREC) under project code NMRR‐19‐1000‐48030.

## CONFLICT OF INTEREST

The authors declare that they have no competing interests.

## AUTHOR CONTRIBUTIONS

FDAN designed the research study, carried out the statistical analysis, and drafted and wrote the manuscript. MKNMK co‐designed the research study and revised the manuscript. SMAA and NAMB carried out laboratory work and data collection. SNAR, JAJ, AH, and YY revised the manuscript. All authors have read and approved the final manuscript.
