# Supplementary material for: Performance comparison of EasyFix G26 and HYDRASYS 2 SCAN for the detection of serum monoclonal proteins
Source: J Clin Lab Anal. 2020 Mar 6;34(6):e23254. doi: 10.1002/jcla.23254 (PMC7307348; doi:10.1002/jcla.23254)
Supplement: Supplementary file 1 [file JCLA-34-e23254-s001.docx]

**SUPPLEMENTARY TABLE**

Limits of agreement, standard deviation, and mean difference values between H2SCAN vs

EFG26 with Bland Altman analysis .

|  |  |  |  |  |  |  |
| --- | --- | --- | --- | --- | --- | --- |
|  |  |  |  | Limits of agreement δ± 1.96 x SD (at 95% confidence interval) | |  |
|  |  |  |  |  |  |  |
|  | Bland Altman Analysis (Mean H2SCAN + EFG26; g/L) |  |  |  |  |  |
|  |  | Mean difference | SD | Lower LOA | Upper LOA |  |
|  | Albumin | 4.70 | 2.55 | -0.31 | 9.70 |  |
|  | Alpha -1 globulins | 0.34 | 0.25 | -0.14 | 0.83 |  |
|  | Alpha -2 globulins | 1.03 | 0.75 | -0.45 | 2.50 |  |
|  | Beta globulins | 2.50 | 1.33 | -0.11 | 5.10 |  |
|  | Gamma globulins | 2.96 | 2.20 | -1.36 | 7.28 |  |
|  | H2SCAN; Hydrasys 2 SCAN, EFG26; Easy Fix G26, SD; Standard Deviation, LOA; Limits of agreement | | | | |  |
|  |  |  |  |  |  |  |
